# HIV-1 and recombinant gp120 affect the survival and differentiation of human vessel wall-derived mesenchymal stem cells

**DOI:** 10.1186/1742-4690-8-40

**Published:** 2011-05-25

**Authors:** Davide Gibellini, Francesco Alviano, Anna Miserocchi, Pier Luigi Tazzari, Francesca Ricci, Alberto Clò, Silvia Morini, Marco Borderi, Pierluigi Viale, Gianandrea Pasquinelli, Pasqualepaolo Pagliaro, Gian Paolo Bagnara, Maria Carla Re

**Affiliations:** 1 Department of Haematology and Oncological Sciences, Microbiology Section, University of Bologna, Italy; 2 Department of Histology, Embryology and Applied Biology, University of Bologna, Italy; 3 Transfusion Medicine Service, St Orsola Hospital, Bologna, Italy; 4 Department of Internal Medicine, Aging and Nephrology, Infectious Diseases Section, University of Bologna, Italy; 5 Department of Haematology, Oncology and Clinical Pathology, University of Bologna, Bologna, Italy; 6 Interuniversity Consortium, National Institute Biostructure and Biosystems (INBB) Rome, Italy

**Keywords:** HIV-1, gp120, mesenchymal stem cells, cell differentiaton, apoptosis

## Abstract

**Background:**

HIV infection elicits the onset of a progressive immunodeficiency and also damages several other organs and tissues such as the CNS, kidney, heart, blood vessels, adipose tissue and bone. In particular, HIV infection has been related to an increased incidence of cardiovascular diseases and derangement in the structure of blood vessels in the absence of classical risk factors. The recent characterization of multipotent mesenchymal cells in the vascular wall, involved in regulating cellular homeostasis, suggests that these cells may be considered a target of HIV pathogenesis. This paper investigated the interaction between HIV-1 and vascular wall resident human mesenchymal stem cells (MSCs).

**Results:**

MSCs were challenged with classical R5 and X4 HIV-1 laboratory strains demonstrating that these strains are able to enter and integrate their retro-transcribed proviral DNA in the host cell genome. Subsequent experiments indicated that HIV-1 strains and recombinant gp120 elicited a reliable increase in apoptosis in sub-confluent MSCs. Since vascular wall MSCs are multipotent cells that may be differentiated towards several cell lineages, we challenged HIV-1 strains and gp120 on MSCs differentiated to adipogenesis and endotheliogenesis. Our experiments showed that the adipogenesis is increased especially by upregulated PPARγ activity whereas the endothelial differentiation induced by VEGF treatment was impaired with a downregulation of endothelial markers such as vWF, Flt-1 and KDR expression. These viral effects in MSC survival and adipogenic or endothelial differentiation were tackled by CD4 blockade suggesting an important role of CD4/gp120 interaction in this context.

**Conclusions:**

The HIV-related derangement of MSC survival and differentiation may suggest a direct role of HIV infection and gp120 in impaired vessel homeostasis and in genesis of vessel damage observed in HIV-infected patients.

## Background

Although the main targets of HIV infection pathogenesis are the CD4+ cells of the immune system, several studies have clearly shown that HIV infection directly and/or indirectly targets other cell lineages and organs [1]. In particular, HIV progressively hampered the homeostasis and functionality of the CNS, bone, kidney and cardiovascular system. These organ-specific lesions have gained a growing importance in the monitoring of HIV infected patients [2-4], especially since the advent of highly active anti-retroviral therapy (HAART) that has increased the patients' life expectancy thereby determining a chronic disease evolution [5].

Clinical and epidemiological studies have shown a consistent connection between HIV infection and a significantly increased incidence of cardiovascular events [6-9], atherosclerosis, coronary arterial disease and pulmonary hypertension [10]. Some reports have clearly demonstrated that HIV infection represents an independent risk factor for atherosclerosis and coronary arterial disease, and atherosclerotic lesions have been observed in coronary, peripheral and cerebral arteries of HIV positive subjects in the absence of classical risk factors [6,11,12]. Carotid artery thickening was up to 24% higher in HIV patients compared with uninfected sex- and age-matched individuals [13-15] and large retrospective studies have proved that HIV positive subjects have a higher incidence of cardiovascular events than uninfected individuals [7,16,17]. These cardiovascular diseases are mainly related to impaired vessel wall homeostasis [18]. In particular, atherosclerosis is linked to severe endothelial dysfunction with arterial wall injury due to factors that trigger a chronic inflammatory response with subsequent atheromatous plaque formation [19,20]. The mechanisms involved in the genesis of atherosclerosis and subsequent cardiovascular damage in HIV positive patients have still not been elucidated, even though some putative indications were recently reported [10].

HIV infection is associated with systemic inflammation and chronic immune activation determining a dysregulation of several cytokines such as IL-6, TNF alpha, M-CSF, IL-10 and IL-1 [21-24]. These cytokines may be involved in the atherosclerosis to different extents, activating and inducing the migration of monocytes in the vessel structures and eliciting the evolution to macrophages [25,26]. Monocytes are known to be the precursors of lipid-laden foam cells within the atherosclerotic plaque [27] producing high levels of pro-inflammatory cytokines thereby determining an inflammatory positive feed-back [10]. Moreover, HIV infection affects cholesterol metabolism especially by viral Nef protein, impairing cholesterol metabolism and cholesterol transport in macrophages and probably hastening the development of vessel structure damage [28,29]. Besides the inflammatory pathway, HIV directly affects endothelial cell layer homeostasis: gp120 and Tat elicit apoptosis in endothelial cells [30-32] through caspase activation. HIV-1 gp120 induces a direct release of endothelin-1, IL-6 and TNFα in endothelial cells leading to direct vessel injury by continuous endothelial damage. Recent observations showed that the homeostasis of the endothelial layer structure does not depend exclusively on circulating endothelial progenitors but can also be regulated by multipotent MSCs [33-36]. MSCs were isolated in the adventitia and in the subendothelial region of vessels and can be differentiated towards several cell lineages such as endothelial cells, osteoblasts, adipocytes and smooth muscle cells [37,38]. Hence, these cells may be the targets of HIV and/or viral proteins inducing direct or indirect vessel damage. To our knowledge, no study has been performed on the interplay between HIV infection and MSCs derived from vascular wall structures to investigate its possible role in the induction of cardiovascular disease and atherosclerosis. The specific studies performed on MSCs and HIV interaction were focused on MSCs or stromal cells isolated from bone marrow [39-43]. These reports described HIV-related bone marrow derangement mechanisms demonstrating that some strains of HIV are able to infect these cells albeit to a low extent [39,40,43] impairing their clonogenic potential with a strong effect on bone marrow cell regulation [40]. In addition, the bone marrow-derived MSCs were affected by viral proteins such as Tat, gp120, Rev and p55 in the specific differentiation to different cellular lineages [41,42]. The aim of our study was to determine the biological effects of HIV infection and gp120 treatment on vascular wall-derived mesenchymal cells to elucidate a possible additional mechanism underlying the vessel dysfunctions observed in HIV-infected patients.

## Materials and methods

### Cell cultures and MSC isolation and differentiation

Human arterial segments of femoral arteries from three male multi-organ heart-beating donors (mean age 39 years) were harvested and used for cell isolation as previously described [38,44]. These vascular artery segments did not have the requirements of length and calibre for clinical use. Isolated MSCs were characterized by flow cytometry and their multi-differentiation potential was determined as previously described [38]. The flow cytometry characterization was carried out on cells taken at passages 3-5 detached by trypsin and washed twice with phosphate-buffered saline (PBS) containing 2% fetal calf serum (FCS; Gibco, Paisley, UK). The cells were stained for 20 minutes at room temperature using the following monoclonal antibodies (mAbs): fluorescein isothiocyanate (FITC) anti-CD29, phycoerythrin (PE)-anti-CD34, FITC-anti-CD44, FITC-anti-CD45, FITC-anti-CD73, PE-anti-CD90, PE-anti-CD105, PE-anti-CD146, PE-anti-CD166 and FITC-anti-KDR, (all from Beckman-Coulter, Fullerton, CA, USA). vWF expression was revealed after permeabilization with the Intraprep Kit (Beckman-Coulter), then incubated with vWFmAb (1/20 in PBS; DakoCytomation, Glostrup, Denmark) for 1 hour at room temperature and subsequently incubated with secondary anti-mouse IgG FITC (1/40 in PBS; DakoCytomation) for 30 minutes at room temperature. PE- or FITC- irrelevant isotype matched mAb served as negative controls. The cells were extensively washed in PBS and then analyzed by Cytomics FC500 Flow Cytometer (Beckman-Coulter). Isolated MSCs were cultured in D-MEM (Lonza, Basel, Switzerland) plus 10% FCS and split every 3-4 days at about 70% density. MSCs were usually seeded at a density of 5 × 10^3 ^cells/cm^2^. For culture expansion, 75 cm^2 ^and 25 cm^2 ^flasks (Becton Dickinson, Palo Alto, CA) treated with collagen (Sigma, St Louis, MO, USA) were used as previously described [44], while for the experiments, the MSCs were seeded in untreated 6-well or 24-well plates (Nunc, Rochester, NY, USA) and employed between passages 4 and 8. To induce adipogenic differentiation, confluent cells were cultured as follows: three cycles of 3 days induction medium and 3 days maintenance medium of hMSC Mesenchymal Stem Cell Adipogenic Differentiation Medium kit (Lonza) were carried out. After a few days the cells containing neutral lipids in fat vacuoles were stained with fresh red oil solution (Sigma) as previously described [45]. MSCs cultured only with adipogenic maintenance medium were taken as the negative control for differentiation. Angiogenic differentiation was assessed on confluent cells, cultured in DMEM (Lonza) with 2% FCS and 50 ng/ml Vascular Endothelial Growth Factor (VEGF; Invitrogen, Carlsbad, CA, USA) for 7 days, changing the medium every 2 days. MSCs cultured in medium without VEGF throughout the induction period were considered the negative control for differentiation [45,46]. NK-92 cells were kept in α-MEM (Gibco) plus 15% FCS, 15% horse serum (Gibco) and 20 U/ml of recombinant human IL-2 (Peprotech, London, UK). Peripheral blood mononuclear cells (PBMCs) were obtained from healthy donors who gave their informed consent following the Helsinki declaration. PBMCs were kept in RPMI 1640 plus 10% FCS or activated by PHA (5 μg/ml; Sigma) plus IL-2 (10 U/ml).

### Viral stocks and infection procedures

HIV-1_IIIB _and HIV-1_Ada _stocks were achieved as previously described [40] and titrated by ELISA HIV-1 p24 antigen kit (Biomerieux, Marcy L'Etoile, France). The heat-inactivated HIV-1_IIIB _(hiHIV-1_IIIb_) and HIV-1_ada _(hiHIV-1_ada_) viruses were obtained after a cycle of inactivation at 65°C for 30 minutes [47]. HIV-1 infection of MSCs was carried out at 50-60% of confluence with HIV-1_IIIB _or HIV-1_Ada _(5 ng/ml of HIV-1 p24) in 6-well or 24-well plates for 2 hours at 37°C. The MSC cultures were extensively washed with PBS, kept in medium and cells and supernatants were harvested at specific times. The HIV-1 p24 content in the infection experiments was assayed by ELISA HIV-1 p24 antigen kit (Biomerieux). In some experiments on sub-confluent MSCs the cell cultures were treated with hiHIV-1 strains (5 ng/ml of HIV-1 p24) or recombinant gp120 (1 μg/ml; NIBSC) for 2 hours at 37°C. As controls, the MSCs were treated with p24 (1 μg/ml; NIBSC) or with HIV-1 strains, hiHIV-1 or gp120 pre-treated for 30 minutes at 37°C with 20 μl of rabbit anti-gp120 pAb (NIBSC, Potters Bar, UK) or, alternatively with 20 μl of rabbit anti-p24 pAb (NIBSC). When confluent MSCs were differentiated to endothelial cells, the same treatment by HIV-1 strains or viral proteins was performed before VEGF stimulation. In the experiments on MSCs differentiated to adipogenesis, HIV-1_IIIB _or HIV-1_Ada _(5 ng/ml of HIV-1 p24), hiHIV-1 strains (5 ng/ml of HIV-1 p24) or recombinant gp120 (1 μg/ml; NIBSC) were added to cell cultures for 2 hours at 37°C before every differentiating medium replacement. At specific times post-treatment, the cells were collected for appropriate molecular and flow cytometry analysis the procedures described below. The CD4 receptor blockade was performed by p5p (Sigma) treatment as described previously [42,48].

### Proviral and integrated DNA detection

Cellular and proviral DNAs were extracted from samples by DNAeasy kit (Qiagen, Hilden, Germany). Purified DNA (0.5 μg) was amplified by PCR using SK431 and SK462 HIV-1 *gag *gene oligos as previously described [49]. A specific amplicon of 142 bp was detectable by 2% agarose gel electrophoresis. As a control, parallel amplification of globin gene was carried out as previously described [50]. The integrated HIV-1 proviral DNA was analyzed after gel purification of cell genomic DNA [51] followed by nested Alu-PCR assay as assessed by O'Doherty and coworkers [52]. The first nested PCR amplification was performed on cell genomic DNA (0.5 μg) with primers specific for *Alu *and *gag *sequences whereas the second amplification was carried out with HIV-1 LTR oligonucleotide pair. A specific amplicon of 100 bp was detectable by 3% agarose gel electrophoresis.

### Qualitative and quantitative RT-PCR amplification

Total mRNA was extracted either from MSCs, PBMCs, NK-92 or from E. coli Dh5α bacteria by High Pure RNA isolation kit (Roche) following the manufacturer's instructions. Total RNA (100 ng) was retro-transcribed and amplified using Quantitect SYBR Green RT-PCR kit (Qiagen) using 400 nM of each β-actin, CD4, CCR5 and CXCR4 specific oligos (for sequences see [49]) in a LightCycler instrument (Roche). The amplification was performed with RT step (1 cycle at 50°C for 20 min) followed by initial activation of HotStar Taq DNA Polymerase at 94°C for 15 min and 40 cycles in three steps: 94°C for 10 s, 60°C for 30 s, 72°C for 60 s. β-actin real time RT-PCR amplification was carried out with an annealing step at 60°C for 15 s and an extension time at 72°C for 25 s. The amplicons were also analyzed in 1.5% agarose gel electrophoresis. The amplification of c-kit, BCRP-1, Oct-4, Notch-1, Sox-2, BMI-1 and β2-microglobulin was assessed following the method described by Pasquinelli and coworkers [38].

To quantify the mRNA expression of several cellular genes involved in the endothelial and adipogenic differentiation, total cellular RNA (100 ng) was retro-transcribed and amplified using Quantitect SYBR Green RT-PCR kit (Qiagen) and 400 nM of each specific oligonucleotide. The amplification was performed with RT step (1 cycle at 50°C for 20 min) followed by initial activation of HotStar Taq DNA Polymerase at 95°C for 15 min and 40 cycles in three steps: 94°C for 10 s, 60°C for 15 s, 72°C for 30 s for C/EBP β, C/EBP δ, adipsin, PPARγ, UCP-1, vWF, KDR whereas for Flt-1 an additional step was added at 78°C for 2 s to analyze the fluorescence. The relative quantifications were performed by specific standard external curves as described [53] and the normalization was performed by parallel amplification of ribosomial 18S as described previously [54]. The specific oligo pairs for adipsin, PPARγ, UCP-1 and ribosomal 18S genes were already published [52], whereas the sequences of C/EBP β, C/EBP δ, vWF, Flt-1 and KDR were:

C/EBPβ: 5' TTCAAGCAGCTGCCCGAGCC 3' and 5' GCCAAGTGCCCCAGTGCCAA 3'

C/EBPδ: 5'-GTGCGCACAGACCGTGGTGA-3' and 5' CGGCGATGTTGTTGCGCTCG 3'

vWF: 5' TAGCCCGCCTCCGCCAGAAT 3' and 5' GTGGGCTGGAGGCCACGTTC 3'

Flt-1: 5'GCCCTGCAGCCCAAAACCCA 3' and 5' CGTGCCCACATGGTGCGTC 3'

KDR: 5'GCGAAAGAGCCGGCCTGTGA 3' and 5' TCCCTGCTTTTGCTGGGCACC 3'

### Apoptosis analysis

The apoptotic cells were analyzed on primary sub-confluent MSCs challenged with HIV-1 strains, hiHIV-1 strains or gp120. The cell cultures were washed with PBS and detached by trypsin at specific times after the treatment start. Apoptotic cells were evaluated as previously described [49]. In brief, the cells were fixed in cold ethanol 70% for 15 minutes at 4°C and after washes in PBS the samples were treated with RNase (0.5 mg/ml; Sigma) and then stained with propidium iodide (50 μg/ml; Sigma). The samples were analyzed by FACScan cytometry (Becton-Dickinson) equipped with an argon laser (488 nm) using Lysis II software (Becton-Dickinson).

### Flow cytometry analysis of cell surface and intracellular markers

Flow cytometry analysis of cell surface CD4, CXCR4 and CCR5 was carried out by FITC-anti-CD4mAb (Becton-Dickinson), FITC-anti-CXCR4mAb (R&D System, Minneapolis, MI) and FITC-anti-CCR5mAb (R& D System) respectively, whereas FITC- irrelevant isotype-matched mAb served as negative controls. These antibodies were used diluted 1/20 in PBS on 1 × 10^5 ^cells for 20 minutes at room temperature. The cells were extensively washed in PBS and then analyzed by Cytomics FC500 Flow Cytometer (Beckman-Coulter). Analysis of intracellular CD4 was performed by staining with the FITC anti-CD4 mAb for 20 minutes at room temperature, after cell fixation with 2% paraformaldehyde and permeabilization with 0.1% saponin. To assay the expression of endothelial specific markers (e.g. Flt-1, KDR, and vWF) by flow cytometry, 1 × 10^5 ^MSCs were analyzed at day 7 after detachment with trypsin. FITC-Flt-1mAb (1/20 in PBS; Santa Cruz Biotechnology, Santa Cruz, CA, USA) and FITC-KDRmAb (R&D System) were used at 1/20 in PBS for 20 minutes whereas to reveal vWF, MSCs were permeabilized with the Intraprep Kit (Beckman-Coulter), incubated with vWFmAb (1/20 in PBS; DakoCytomation) for 1 hour at room temperature and subsequently incubated with secondary anti-mouse IgG FITC (1/40 in PBS; DakoCytomation) for 30 minutes at room temperature. Fluorescence intensity data of intracellular and surface proteins were acquired using a Cytomics FC500 Flow Cytometer (Beckman-Coulter). Results were analyzed using the CXP Software (Beckman-Coulter).

### PPARγ activity assay

PPARγ transcription factor activity was detected by TransAM PPARγ kit (Active Motif, Carlsbad, CA, USA) as indicated by the manufacturer. This approach is a highly sensitive ELISA assay that provides, after the extraction of nuclear proteins, the determination of PPARγ binding on specific consensus sequence fixed on plate wells. This binding was targeted by specific anti-PPARγ mAb revealed by means of an HRP-conjugated secondary pAb and a colorimetric substrate. The assay was read by spectrophotometer at 450 nm and compared with reference curve after protein concentration normalization.

### Statistical analysis

The data are expressed as means ± standard deviation (±SD) of three separate experiments performed in duplicate. Statistical analysis was performed using Student's two-tailed t-test.

## Results

### Human MSCs can be isolated and purified from peripheral artery vascular wall

**Figure 1:**
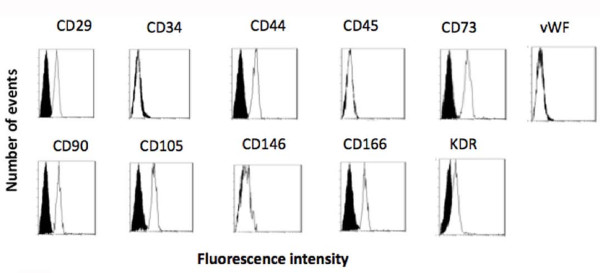
**Analysis of typical MSC markers by flow cytometry**. Shadowed areas represent MSCs treated with fluorochrome-conjugated irrelevant isotype matched mAb, whereas unshadowed areas are the MSCs stained with specific fluorochrome-conjugate mAb. A typical pattern of CD29, 34, 44, 45, 73, 90, 105, 146, 166, vWF, KDR is shown.

**Figure 2:**
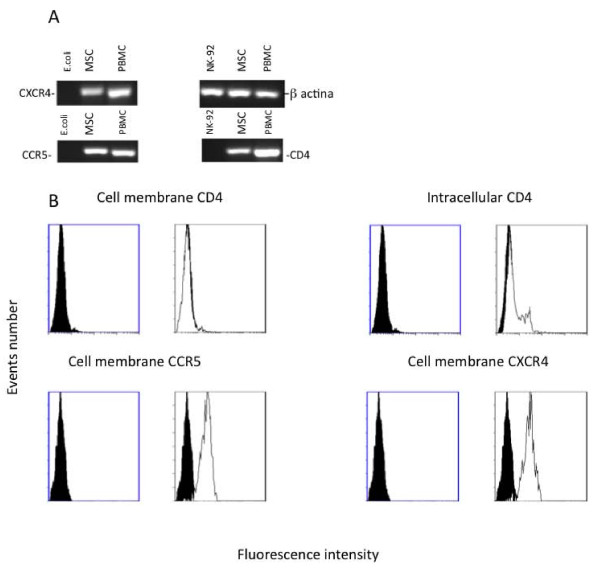
**Analysis  of  CD4,  CXCR4  and  CCR5  expression  in  MSCs. **Analysis of CD4, CXCR4, CCR5 and β-actin mRNA expression by qualitative real time RT-PCR in MSCs (A). A typical gel electrophoresis of qualitative real time RT-PCR is shown. As positive controls, total RNAs extracted from PBMC were employed. The total RNAs extracted from NK-92 cells (for CD4) and *E. coli *total RNA (for CXCR4 and CCR5) served as negative control. Panel B displays a typical flow cytometry analysis of CCR5, CXCR4 and CD4 staining in MSCs. Unshadowed areas represent MSCs treated with FITC-conjugated specific mAb, whereas the negative control (MSCs stained by FITC-conjugated irrelevant isotype matched mAb) is represented by shadowed areas. Three experiments were performed in duplicate.

Human vascular wall-derived MSCs were characterized by cellular and molecular approaches. Flow cytometry analysis showed that these cells expressed a reliable cell marker phenotype with CD29+, CD44+, CD73+, CD90+, CD105+, CD166+, KDR^low^, CD34^-^, CD45^-^, CD146^- ^and vWF^- ^(Figure 1). Parallel molecular analysis showed that in the early culture passages these cells exhibited RT-PCR positive detection of embryonic stem cell marker Oct-4 as well as some molecules known to play a role in critical regulatory pathways of stem cells, such as c-kit, BCRP-1, Notch-1, Sox-2 and BMI-1 (data not shown). To determine whether these cells also expressed the mRNAs of classical HIV receptor CD4 and co-receptor CXCR4 and CCR5, total RNA was extracted from MSCs and analyzed with the RT-PCR technique. The CD4, CXCR4 and CCR5 mRNAs were currently detectable as shown in Figure 2A. In parallel, the expression of CD4, CXCR4 and CCR5 proteins was analyzed on the cell membrane using a flow cytometry procedure. CXCR4 and CCR5 were clearly detected on the cell membrane. Staining with FITC-conjugated anti-CD4mAb failed to disclose CD4 protein expression on the cell surface, but when the MSCs were fixed and permeabilized with saponin an intracellular positivity was clearly displayed in about 20% of the cells (Figure 2B). This finding may suggest a complex pattern of CD4 protein regulation expression in these cells that did not rule out the possible presence of a very low level of CD4 protein on the cell membrane below the sensitivity level of flow cytometry.

### HIV-1_ada _and HIV-1 _IIIb _integrate their retrotranscribed proviral DNA in host MSC genome

**Figure 3:**
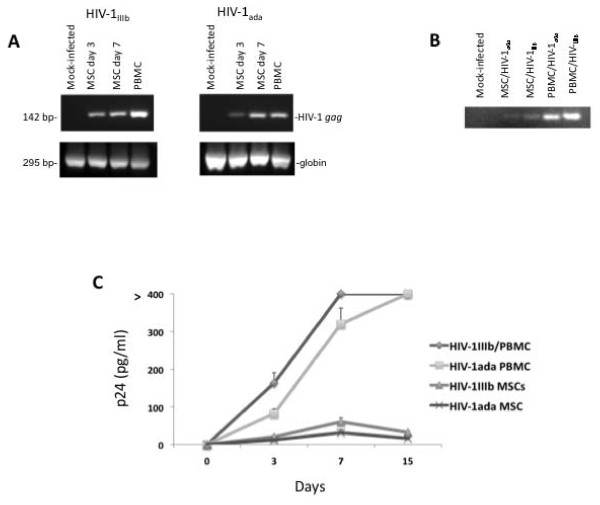
**HIV-­‐1  proviral  DNA  and  p24  protein  detection  in  MSCs  infected  by  HIV-­‐1  strains.** Analysis of HIV-1 proviral DNA by qualitative real time PCR (A): agarose gel electrophoresis of MSC infected by HIV-1_IIIb _and HIV-1_ada _at days 3 and 7 post-infection. Positive control was activated PBMC at day 3 and negative control was mock-infected MSCs. All experiments were performed using 5 × 10^5 ^MSC or activated PBMCs infected or not with HIV-1_IIIb _and HIV-1_ada _(5 ng/ml p24). Panel B shows DNA integrated proviral HIV-1. The total DNA extracted from 5 × 10^5 ^MSC or activated PBMC cells was run in agarose gel electrophoresis and, after the purification of cellular DNA as previously described (**51**), a nested *Alu*-PCR was performed. The MSCs challenged by HIV-1 strains were analyzed at day 7. Activated PBMCs infected with the two HIV-1 strains served as positive controls. A specific LTR 100 bp band is detectable in HIV-1 infected MSCs and positive controls. Panel C displays the cell supernatant p24 analysis. ELISA p24 kit was employed to analyze the p24 content in cell supernatant. This assay exhibits a sensitive limit at 3 pg/ml. The amount of p24 in MSCs challenged with HIV-1 strains was very low and, in these experimental conditions, slowly declined at later tested times. The positive controls were performed by activated PBMC infected with HIV-1 strains.

To determine whether MSCs can be considered targets of HIV-1 infection, subconfluent MSCs were challenged with two classical HIV-1 X4 and R5 laboratory strains represented by HIV-1_IIIb _and HIV-1_ada _respectively. Total DNA, collected and purified at days 3 and 7 post-infection, was analyzed by PCR, and both HIV-1_IIIb _and HIV-1_ada _proviral DNAs were disclosed (Figure 3A). In parallel experiments, the integrated viral DNA in the MSC genome was analyzed by a nested-*Alu *PCR where the first oligo pair amplifies regions of different length between *Alu *regions and HIV-1 gag gene whereas the second amplification was performed with internal HIV-1 specific oligos to obtain a specific 100 bp amplicon. Whole DNA was extracted from MSCs at days 7 and 10 post-infection, and HIV-1 specific 100 bp product was detected (Figure 3B). Hence, these results indicate that both HIV-1 strains enter MSC cells and retrotranscribe their RNA genome to proviral DNA integrating it in the host cell genome. To establish whether HIV infection of MSCs determines the production of new viral progeny, we analyzed the p24 protein burden by ELISA in MSC supernatants. The p24 protein was barely detected and progressively decreased over time suggesting that the MSCs showed a very low permissivity to HIV infection in these experimental conditions (Figure 3C).

### HIV-1 strains and recombinant gp120 induce apoptosis in subconfluent MSCs

**Figure 4:**
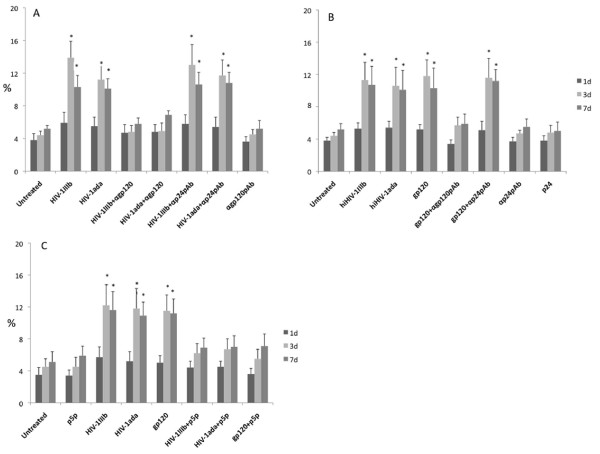
**Determination of apoptotic cell percentage by a flow cytometry procedure**. Sub-confluent MSCs treated with HIV-1 strains (5 ng/ml p24) and recombinant gp120 (1 μg/ml) were assayed (panel A) with propidium iodide staining after cell fixation at different times (days 1, 3, and 7). Panel B reports the apoptosis induction when hiHIV-1 strains or gp120 were used. Panel C represents the apoptotic cell percentages obtained when CD4 blockade by p5p treatment was performed. Statistical significance was determined using Student's t test with *p < 0.05.

Besides the direct infection of specific targets, HIV employs several pathogenetic mechanisms among which apoptosis activation plays a pivotal role in several cell models such as CD34+ hematopoietic progenitor cells and T cells. To investigate whether the interaction between HIV-1 and MSCs induces apoptosis activation, subconfluent MSCs were exposed to both HIV-1 strains, and the apoptotic cell percentage was assessed with propidium iodide flow cytometry technique. The flow cytometry analysis performed at day 1, 3 and 7 post-infection showed a significant increase in apoptotic cells (Figure 4A) in the samples challenged with the two HIV-1 strains at day 3 (13.9 ± 3.2% and 11.2 ± 2.5% in the HIV-1_IIIb _or HIV-1_ada _infected samples respectively, in comparison with 4.4 ± 0.5% of apoptotic cells detected in the mock-infected cultures; p < 0.05) and to a lesser extent at day 7 (10.3 ± 1.4% and 10.1 ± 1.2% in the HIV-1_IIIb _or HIV-1_ada _infected samples respectively, in comparison with 5.2 ± 0.4% in the mock-infected cultures; p < 0.05). The parallel challenge of MSCs with recombinant viral gp120 (11.8 ± 2% vs 4.4 ± 0.5% at day 3) or heat inactivated (hi)HIV-1 strains displayed a similar apoptosis increase pattern (Figure 4B). The pre-treatment of HIV-1 strains or gp120 with neutralizing rabbit pAb to gp120 elicited a clear inhibition of apoptosis induction (Figure 4B). Since the interaction between gp120 and CD4 was related to programmed cell death in different cell models, MSCs were treated by p5p (a CD4 antagonist) and challenged with HIV-1_IIIb_, HIV-1ada or gp120. This p5p treatment induces a significant inhibition of HIV related apoptosis induction at days 3 and 7 indicating that CD4 blockade tackled the HIV-1 and gp120 related MSC apoptosis (Figure 4C).

In the next series of experiments, we studied whether HIV-1 strains and/or gp120 elicited apoptosis in MSCs differentiated towards adipogenic and endothelial cell lineages. Interestingly, biologically active or hiHIV-1 strains and gp120 failed to determine a significant apoptosis induction during the adipogenetic or endothelial differentiation (data not shown) suggesting that these differentiation stimuli could prevent the negative survival signal induced by viral treatment.

### HIV-1 and recombinant gp120 positively modulate the MSCs differentiation to adipogenesis

**Figure 5:**
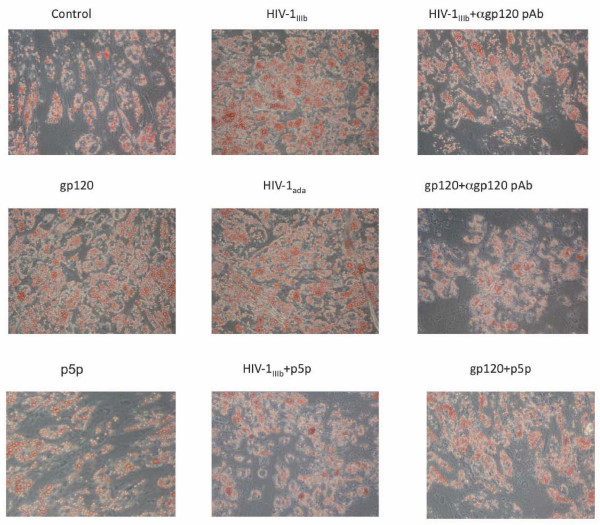
**Red oil staining of MSCs differentiated towards adipogenesis at day 10**. MSCs challenged with HIV-1 strains (5 ng p24/ml) or gp120 (1 μg/ml) displayed more abundant multivacuolar adipogenic vescicles in the cytoplasm than untreated differentiated cells. Neutralizing anti-gp120 pAb or p5p treatment in MSC samples challenged with HIV-1 or gp120 inhibited the increase in red oil stained lipid drop amount. Magnification 200X.

**Figure 6:**
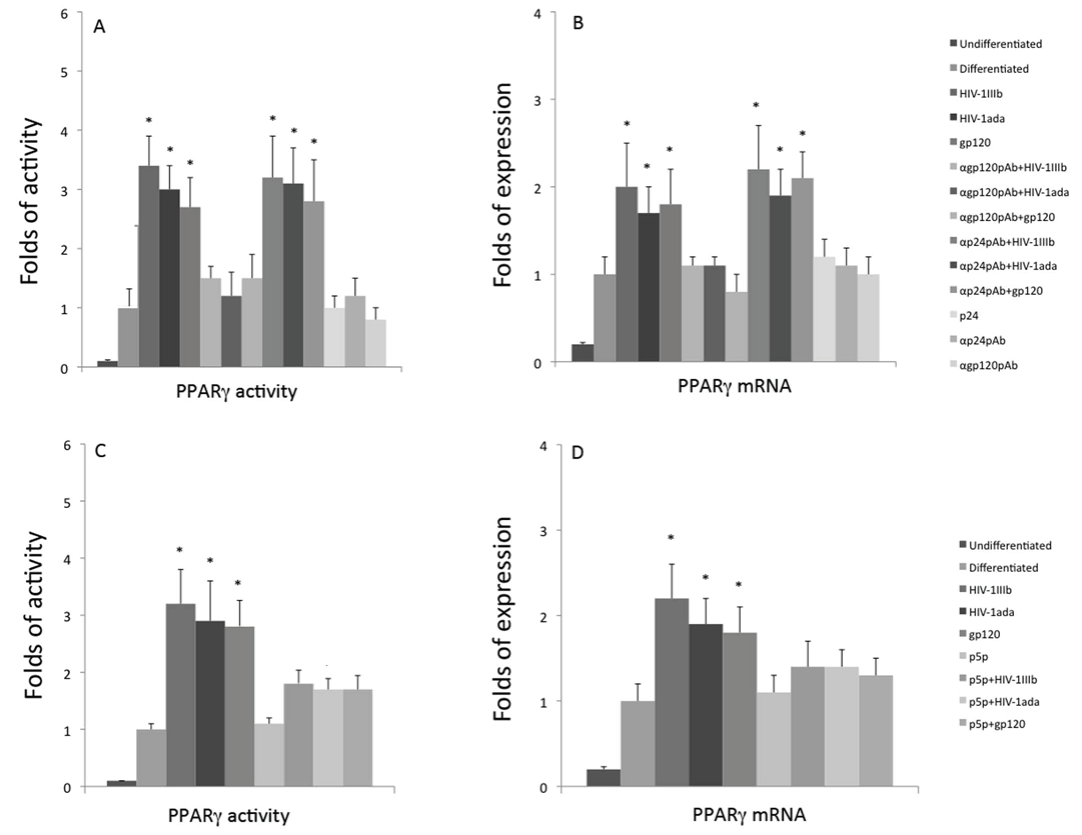
**Effect of HIV-1 strains and gp120 on PPARγ transcription factor activity and mRNA expression in MSCs differentiated to adipogenesis**. In A and C, MSCs were challenged with HIV-1 strains (5 ng/ml) and gp120 (1 μg/ml) in the presence or absence of anti-gp120 pAb, anti p24 pAb and p5p. MSCs were harvested at day 7 and nuclear extracts were processed for PPARγ activity using TransAM PPARγ kit. The PPARγ activity data were expressed by the ratio (±SD) between samples and the control represented by MSC cell cultures differentiated to adipogenesis. The adipogenesis differentiated cell culture PPARγ activity was set at 1. Three experiments performed in duplicate were carried out. In B and D, quantitative real-time RT-PCR was performed at day 7 to analyze PPARγ mRNA expression in cell cultures treated with the viral strains and viral proteins. The mRNA expression data were expressed by the ratio between samples and the control represented by adipogenesis differentiated cell cultures after 18S ribosomial normalization. The adipogenesis differentiated cell culture mRNA was set at 1. Three experiments performed in duplicate were carried out. Statistical significance was determined using Student's t test with *p < 0.05.

**Figure 7:**
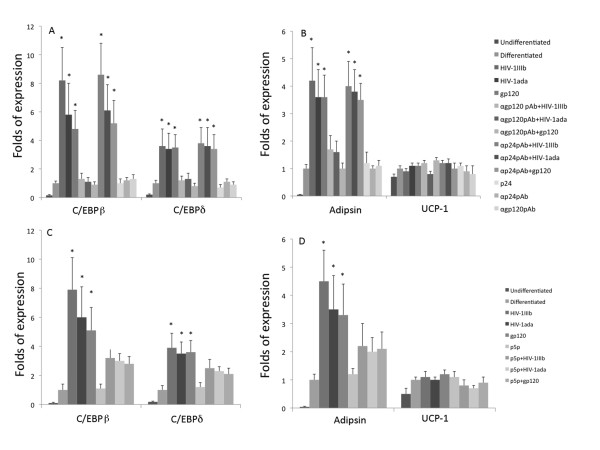
**Analysis of mRNA expression of specific early and late genes involved in adipogenesis by quantitative real time RT-PCR**. MSCs were challenged with HIV-1 strains (5 ng/ml) and gp120 (1 μg/ml) in the presence or absence of anti-gp120 pAb, anti p24 pAb and p5p. Panel A and C show the up-regulation of two transcription factor gene expressions such as C/EBP β and C/EBP δ involved in the early stages of differentiation. The analysis was performed at day 3 post-stimulation and the two mRNA were significantly increased with respect to adipogenesis differentiated cell cultures. In B and D, some late markers of adipogenic differentiation such as adipsin and UCP-1 mRNA expression were analyzed. Adipsin was significantly up-regulated at day 10 whereas UCP-1 a marker of brown fat was barely expressed and without any significant difference with respect to the controls. The results were expressed by the ratio between samples and the control represented by adipogenesis differentiated cell cultures after 18S ribosomial normalization. The adipogenesis differentiated cell culture mRNA was set at 1. The data represent the mean (+SD) of three independent experiments performed in duplicate. Statistical significance was determined using Student's t test with *p < 0.05.

MSCs isolated from blood vessels can be differentiated into several lineages such as osteoblast, adipocyte, smooth muscle and endothelial cells. To study the effects of HIV-1 on the differentiation of these cells, the interaction of HIV-1 and recombinant gp120 on MSC differentiation to adipogenic and endothelial lineages was analyzed. The adipogenic differentiation was tested at different times by direct staining of cell cultures with red oil. The microscopic evaluation of the red oil stained cell cultures showed a reliable increase in red oil stained cells in the cell cultures treated with viral agonists at days 7 and 10 (Figure 5), in comparison with control cultures indicating that the HIV-1 and gp120 enhanced a more rapid and massive differentiation of MSC stimulated to adipogenic lineage. Since PPARγ is currently considered the most important regulator of adipogenesis through its transcription factor activity, we assayed with ELISA TransAM assay the PPARγ activity at day 7 in the same experimental conditions. HIV-1_IIIb_, HIV-1_ada _and recombinant gp120 induced (Figure 6A) a significant up-regulation of PPARγ activity in comparison with the cell culture control (3.4 ± 0.5 fold increase with HIV-1_IIIb _(p < 0.05), 3 ± 0.4 fold increase with HIV-1_ada _(p < 0.05) and 2.7 ± 0.5 fold increase with gp120 (p < 0.05) when the cell cultures were challenged either by HIV-1 strains or gp120. This effect was abolished when HIV-1 strains or gp120 were pre-treated with anti-gp120 pAb. In parallel, the PPARγ mRNA content evaluated by quantitative real time RT-PCR (Figure 6B) showed a slight but significant up-regulation of specific transcripts (2 ± 0.5 fold increase with HIV-1_IIIb_; p < 0.05, 1.7 ± 0.3 fold increase with HIV-1_ada_; p < 0.05 and 1,8 ± 0,4 fold increase with gp120; p < 0.05) with respect to induced cell culture controls. Since adipogenesis is regulated by several factors modulating specific gene expression, the mRNA expression of other specific genes involved in adipogenesis regulation was analyzed. The early steps of differentiation are linked to activation of C/EBP β and δ, which, in turn, activate C/EBP α and PPARγ inducing the complete differentiation to mature adipocyte with the expression of late differentiation markers such as adipsin and UCP-1. The analysis of C/EBP β and δ mRNA expression was analysed by quantitative real time RT-PCR (Figure 7A). HIV-1 and gp120 induced a significant up-regulation of C/EBP β (8.2 ± 2.3, p < 0.05 with HIV-1_IIIb_, 5.8 ± 1.4 p < 0.05 with HIV-1_ada _and 4.7 ± 1.3 p < 0.05 with gp120) and δ (3.6 ± 1.2, p < 0.05 with HIV-1_IIIb_, 3.4 ± 1.3 p < 0.05 with HIV-1_ada _and 3.5 ± 0.9 p < 0.05 with gp120) mRNAs at day 3. As expected, the pre-treatment of HIV-1 strains or gp120 with anti-gp120 pAb inhibited the specific mRNA increase. In parallel, some late adipogenetic markers such as adipsin and UCP-1 mRNAs expression were studied with quantitative real time RT-PCR at day 10. HIV-1 strains and gp120 positively modulated the adipsin mRNA expression whereas UCP-1 is poorly expressed and did not show any significant quantitative mRNA variation related to any treatment (Figure 7B) suggesting that MSCs in these experimental conditions underwent a differentiation toward white fat rather than brown fat. The CD4 blockade by p5p determined a significant decrease of adipogenesis induction (Figure 5) by HIV-1 strains and gp120 as well as PPARγ activity up-regulation (Figure 6C-D). Consistently, the treatment with p5p also decreased the HIV-related activation of C/EBPβ, C/EBPδ and adipsin mRNA expression (Figure 7C-D) indicating a general down-regulation of HIV related proadipogenetic effects.

### HIV-1 and recombinant gp120 inhibit the MSCs endothelial differentiation

**Figure 8:**
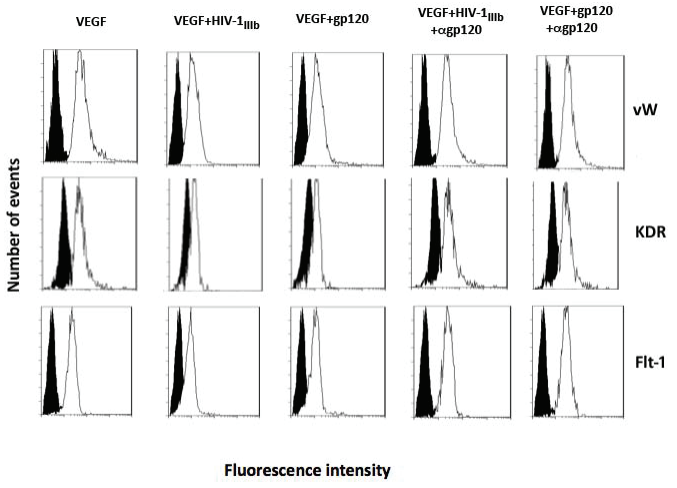
**HIV-1 strains and gp120 inhibited the protein expression of some specific differentiation markers on MSCs differentiated towards the endothelial lineage**. MSCs were differentiated to endothelial cells by VEGF treatment and at day 7 the cell cultures were collected and flow cytometry analysis of vWF, Flt-1 and KDR protein showed an inhibition of these three markers in MSC samples challenged with HIV-1 or gp120. In the histograms, shadowed areas represent the isotype irrelevant FITC-labeled mAb treated samples, the unshadowed areas represent VEGF treated sample challenged with HIV-1 or gp120 with or without neutralizing anti-gp120 pAb. A typical experiment is shown.

**Figure 9:**
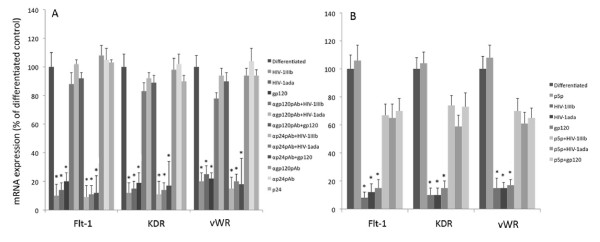
**Determination of vWF, Flt-1 and KDR mRNA by quantitative real-time RT-PCR**. MSCs were differentiated to endothelial cells by VEGF and challenged with HIV-1 strains and gp120 in the presence or absence of anti-gp120 pAb, anti p24 pAb (panel A) and p5p (panel B). Total RNA was extracted and purified at day 7 and vWF, Flt-1 and KDR mRNAs were analysed. The results were expressed by the ratio between samples and the control represented by VEGF-differentiated cell cultures after 18S ribosomial normalization. The VEGF treated control cell culture mRNA was set at 100. The data represent the mean (+SD) of three independent experiments performed in duplicate. Statistical significance was determined using Student's t test with *p < 0.05.

In a next series of experiments, we investigated the effects of HIV-1 and gp120 on endothelial differentiation of MSCs induced by VEGF treatment. When MSCs were treated with VEGF, they differentiated to endothelial cells exhibiting several specific endothelial markers. To assess whether the endothelial differentiation may be positively or negatively affected by viral challenge, the expression of some endothelial markers such as vWF, Flt-1 and KDR was analyzed by a flow cytometry procedure. This approach displayed a clear decrease of all three markers (Figure 8) when MSCs were challenged by gp120 or HIV-1 strains. In parallel, quantitative real time RT-PCR was carried out and the results confirmed a significant decrease of Flt-1, KDR and vWF mRNA expression when HIV-1 strains or gp120 were added to cell cultures (p < 0.05; Figure 9A). Moreover, the treatment of cell cultures with p24 or gp120 and HIV pre-treated with neutralizing anti-gp120 pAb did not show any significant biological effect on mRNA and protein endothelial marker expressions.

**Figure 10:**
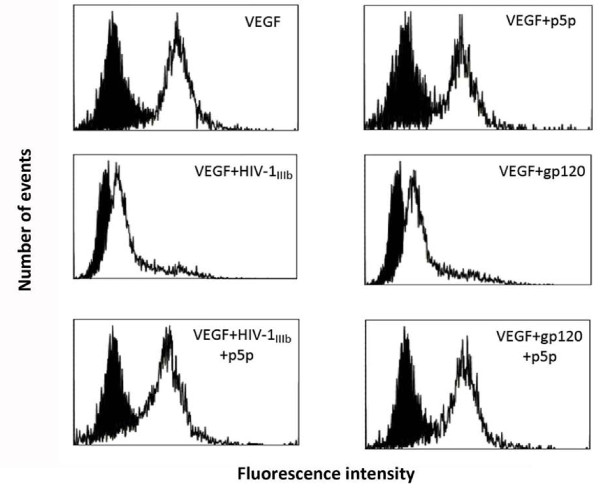
**Flow cytometry analysis of vWF intracellular protein in MSC challenged by HIV-1 and gp120 with or without p5p treatment at day 7**. CD4 blockade by p5p inhibited the HIV-related negative modulation of intracellular vWF protein in VEGF-differentiated cell cultures. Shadowed areas represent samples treated with irrelevant mAb plus FITC-conjugated secondary antibody, whereas unshadowed areas are the MSCs stained with anti-vWF mAb plus FITC-conjugated secondary antibody. A typical experiment is shown.

We also analysed whether p5p treatment may affect the HIV-related inhibition of VEGF-driven MSC differentiation. As shown in Figure 9B and 10, CD4 blockade determined a clear recovery of vWF protein and mRNA expression at day 7 as well as for Flt-1 and KDR (Figures 9 and 10 and data not shown) in HIV-1 and gp120 treated samples.

## Discussion

Human MSCs are multipotent cells that can be isolated from almost all tissues and organs in the human body [55]. These cells have the potential to differentiate by specific stimuli to different cell lineages and can be involved in tissue repair and homeostasis [56]. Several studies have demonstrated MSCs in the blood vessel wall that can be differentiated to endothelial, adipocyte, osteoblast and smooth muscle cells [38,57-59]. In particular, MSCs isolated from the blood vessel wall are strongly involved in the control of endothelial layer structure and vessel wall homeostasis [60] suggesting that their impairment may play an important role in vessel damage and atherosclerotic evolution. Since cardiovascular lesions, particularly atherosclerosis, represent major clinical manifestations during the evolution of HIV-related disease [13-15,61], we investigated the interaction between HIV-1 and MSCs isolated from the vessel wall to establish whether HIV-1 is able to impair MSC biology. This report focused on two main aspects: the direct effects of HIV-1 challenge and gp120 treatment on primary vessel wall MSCs and the impact of viral action on MSC differentiation towards specific cellular lineages to identify possible mechanisms involved in the derangement of vascular structure observed in HIV-1 positive individuals. Two classical HIV-1 X4 and R5 laboratory strains were able to enter, retro-transcribe and integrate the proviral DNA in the MSC host genome but the very low level of p24 protein content detected in the cell supernatant raised the possibility that the MSCs may be considered barely permissive to HIV-1 infection.

Previous studies [39,40,43] disclosed that HIV productively infected bone marrow mesenchymal or stromal cells to different extents. Recent data on bone marrow-derived MSCs demonstrated that HIV-1 proviral DNA integration was detected after HIV-infected sera challenge [42] in the absence of detectable HIV-1 p24 in the cell supernatants. It is conceivable that the relative discordance among the results described in these studies is related to both MSC culture isolation purity and the specific anatomical origins (bone marrow and vessel wall) that may induce different responses to HIV-1 challenge. Interestingly, the indication that vessel wall MSCs were subjected to proviral DNA integration in the DNA host genome may suggest a possible role of MSCs as a potential infection reservoir. However, the importance and the relative possibility of HIV reactivation by this reservoir must be assessed by further studies to discern its true extent and biological impact *in vivo*. Following these data on the sensitivity of MSCs regarding the HIV infection, we also studied the effects of HIV on the survival of primary MSCs. Apoptosis activation plays a pivotal role in some HIV-1-related pathogenetic aspects related to specific cell lineage progressive loss [62]. Programmed cell death is considered an important pathway involved in the progressive decline of CD4+ T lymphocytes and in the anemia, granulocytopenia and thrombocytopenia, due to impaired CD34+ hematopoietic progenitor survival, occurring in several patients during HIV-related disease development [1,2,62]. Moreover, Tat and gp120 are involved in the apoptosis of neuronal and osteoblast cells, respectively, supporting, at least in part, the AIDS dementia complex and the osteopenia/osteoporosis observed in several HIV-positive individuals [3,4,49]. The treatment of sub-confluent vessel wall MSCs with both HIV-1 strains lead to significant apoptosis activation. Interestingly, HIV-1 strains and gp120 are able to elicit apoptosis induction that is inhibited in presence of anti gp120pAb or p5p treatment. This suggests that the interaction between gp120 and CD4 plays an important role in the activation of programmed cell death. HIV-1 gp120 recognizes CD4 as its main receptor even though it is well known to bind other cell receptors such as the galactocerebroside molecule (GalC) determining a wide array of biological effects from infection of susceptible cells to induction of signal transduction intracellular pathways [63]. In particular the interaction between gp120 and CD4 determines apoptosis activation in several cell lineages such as CD34+ hematopoietic progenitor cells and CD4+ cells [64-66]. The vessel wall MSCs express the CD4 mRNA in the absence of detectable amounts of CD4 protein on the cell membrane by flow cytometry analysis. However, the presence of CD4 protein below the sensitivity limit of the technique cannot be ruled out because flow cytometry showed its detection limit at around 1,000 fluorescent molecules [67]. Moreover, the intracellular detection of a low amount of CD4 in about 20% of MSCs suggests a possible complex regulation of CD4 protein expression in these cells. It is noteworthy that this pattern of CD4 expression (mRNA positivity and protein undetectable on cell membrane by flow cytometry) was previously observed on MSC purified from bone marrow [39] and in other cell lines sensitive to HIV infection that underwent productive infection and/or apoptosis induction [67-69]. Interestingly, apoptosis activation was not detected when the MSCs were committed to fat or endothelial cells. The treatment with differentiation inducers and the cell confluence may tackle the HIV-1 strains/gp120-induced negative signals. VEGF, for example, induces a strong activation of cell survival pathways with the phosphorylation of AKT via activation of PI-3-kinase that determines cell survival during the differentiation [70,71]. In addition, MSCs differentiate when the cells are confluent suggesting a possible role of the cell cycle and then a specific pattern of transcription factors in survival regulation.

Since the vessel wall MSCs exhibited cell differentiation multipotency, we analyzed the HIV-1 impact on MSCs when these cells were differentiated towards specific cell lineages represented by adipocytes and endothelial cells. Adipogenesis is regulated through a sequence of cellular and molecular events well described in pre-adipocye cell models such as the 3T3-L1 cell line and stem cell lines [72,73]. After the growth arrest in confluence, the cells in these models were subjected to clonal expansion mediated to induction of C/EBP β and C/EBP δ [74,75] that positively regulate the expression of some adipocyte specific genes. In particular, these transcription factors activate C/EBPα and PPARγ [76], which in turn modulate the further steps of the differentiation programme to adipocytes. PPARγ is a pivotal factor for in vivo adipogenesis: PPARγ deficient mice are characterized by a total absence of white and brown adipose tissue [77]. In vessel wall MSCs, HIV-1 and gp120 are able to enhance adipogenesis and up-regulate PPARγ activity. PPARγ has already been described as a target of gp120. Cotter and coworkers [78] reported increased PPARγ activation in primary osteoblasts with a dysregulation of osteoblastogenesis also associated with RUNX-2 inhibition. In addition, Rev and p55 were able to activate PPARγ in MSCs from bone marrow [41]. These observations suggested that the modulation of PPARγ by HIV proteins during HIV infection may be considered a co-factor in the lipid and bone derangement observed during the HIV infection and in some antiretroviral treatments [79]. Our data also showed that viral infection and gp120 exposure induced an up-regulation of C/EBPβ and δ mRNA expressions demonstrating that adipogenesis positive modulation is determined until the first differentiation molecular steps. Interestingly, C/EBPβ factor modulates HIV-1 expression and replication in monocyte/macrophages and is even activated by the gp120/CD4 interaction through the MAP kinase pathway [80] suggesting a more complex role of this transcription factor in HIV-1 pathogenesis. Of note, CD4 blockade determined a significant decrease of HIV-related adipogenesis in agreement with data described by Cotter and coworkers in bone marrow-derived MSCs (**42**). Altogether, these observations suggest that these HIV-related pro-adipogenic effects in MSCs purified from different anatomical districts, might be induced, at least in part, by CD4/gp120 binding.

Until the study by Asahara and coworkers [33], it was generally postulated that the formation of new vessels in the adult originated from sprouting of pre-existing vessels [81,82]. More recent studies showed that endothelial cells could also be renewed by other cell types such as mesenchymal cells with a more complex regulation of vessel wall structure homeostasis [36,37,83]. In particular, adult human arteries contain multipotent MSCs that reside within specific zones of the vascular wall such as the sub-endothelial space and vascular adventitia [37]. In our model, we analyzed the impact of HIV-1 and gp120 protein on MSCs differentiated by VEGF treatment to endothelial cells studying the mRNA and protein expression of specific endothelial markers such as vWF, Flt-1 and KDR. Unlike adipogenesis, endothelial differentiation was impaired by HIV-1 and gp120 as documented by a decrease of vWF, Flt-1 and KDR mRNA and protein expression that is strongly inhibited by CD4 blockade. This impairment of endothelial differentiation may represent an interesting additional pathogenetic model in HIV-1 infection. The dysfunction of endothelial cells and vascular structure has been proven in HIV patients [9,10]. In particular, several studies have described how chronic inflammation, platelet activation and hypercoagulability elicit damage to endothelial homeostasis [84-86] and may play a role in the linkage between HIV infection and cardiovascular disease [9].

HIV-related chronic inflammation determines the expression of several cytokines especially in mononuclear cells such as IL-6, IL-8 and TNF α [22,23,87,88] which activate endothelial cells and enhance leukocyte adhesion. In addition, gp120 is able to determine direct and indirect injury to endothelial cells. This viral glycoprotein induces apoptosis in endothelial cells especially in the lung, thereby contributing to pulmonary hypertension [30,89]. HIV-1 gp120 also stimulates the activation of cytokines like IL-6 and TNF α [90] resulting in damage to endothelial vessel structure.

Vascular injury and atherosclerosis have been described in HIV positive patients: autopsy reports demonstrated atherosclerotic lesions in peripheral, coronary and cerebral arteries in the absence of traditional atherosclerosis risk factors [11-13]. Several retrospective studies performed on HIV-infected individuals have demonstrated a two-three fold rise in the incidence of cardiovascular disease in comparison with sex and age-matched healthy subjects [7,16,61]. In addition, an analysis of surrogate markers of coronary artery disease showed that carotid artery intima-media thickness (IMT) is increased up to 24% in HIV-infected patients with respect to controls [13].

The HIV-related mechanisms of atherosclerosis induction and cardiovascular damage remain unsettled. Some studies have suggested that the atherosclerosis induction observed in HIV-positive patients is linked to the direct effect of HIV infection and/or viral proteins on cholesterol metabolism in monocytes [28,91,92]. These cells represent the precursors of the lipid foam cells within the atherosclerotic plaque producing a high level of IL-6, a cytokine positively regulated also by Tat [10,21,24]. The foam cells produce proatherogenic factors such as chemokines, cytokines and metalloproteinases, which promote plaque expansion with instability of lesions and vascular cell degeneration and apoptosis [10,29]. In addition, chronic infection and endothelial damage determine a significant increase in monocyte migration exacerbating vessel damage [25].

## Conclusions

Our data suggest an additional HIV-related mechanism of vessel wall and endothelial layer injury, involving MSCs. HIV affects MSC biology acting on both uncommitted and committed MSCs. HIV is able to integrate its genome in vascular wall MSC DNA. Apoptosis activation is mainly activated through the gp120/CD4 interaction that also plays an important role even in differentiation derangement. This shows a direct effect on primary MSCs that decreases the viable MSCs suited for vessel structure homeostasis. Our findings may even suggest the role of reservoir for HIV infection in MSCs although the true clinical and virological impact awaits clarification.

HIV and gp120 negatively influence endotheliogenesis thereby facilitating diffuse vascular damage and the promotion and development of atherosclerosis lesions due to impaired MSC repair control of endothelial and vessel structure. On the other hand, HIV and gp120 induce the differentiation of MSCs to adipocytes through PPARγ activity and C/EBP β expression up-regulation leading to speculate that not all subintimal foam cells originate from monocytes. The HIV-related induction of adipocyte differentiation can determine a derangement of MSC differentiation balance with a possible involvement in atherosclerosis genesis and development. Interestingly, some peculiar lesions are found in atherosclerothic vessel degeneration such as cartilaginous metaplasia with endocondral ossification and fat tissues, especially in inflammatory abdominal aortic aneurysms [93,94] that have not been well explored and might be related to dysregulated MSC differentiation. Altogether, these results indicate that HIV and gp120 have a strong direct impact on vessel wall MSC biology and differentiation. These observations may help to explain the early and diffuse atherosclerosis and vascular damage observed in HIV-infected patients.

## Conflict of interest statement

The authors declare that they have no competing interests.

## Authors' contributions

DG and MCR conceived the study, DG, MCR and FA designed the study, DG, FA, PLT, FR and PP performed flow cytometry analysis, FA, GPB and GP isolated the MSCs, DG, FA, AM, AC, SM carried out cell differentiation, infection and molecular experiments, MB, PV and DG performed apoptosis analysis, DG drafted the manuscript, FA and MCR reviewed it, All authors contributed to the final version of manuscript, read and approved it.

## References

[B1] Levy JA HIV pathogenesis: 25 years of progress and persistent challenges AIDS 2009 23 147 160 10.1097/QAD.0b013e3283217f9f 19098484

[B2] Fauci AS HIV and AIDS: 20 years of science Nat Med 2003 9 839 843 10.1038/nm0703-839 12835701

[B3] Mattison MP Haughey NJ Nath A Cell death in HIV dementia Cell Death Differ 2005 12 893 904 15761472 10.1038/sj.cdd.4401577

[B4] Borderi M Gibellini D Vescini F Biagetti C De Crignis E Re MC Metabolic bone disease in HIV infection AIDS 2009 23 1297 1310 10.1097/QAD.0b013e32832ce85a 19550284

[B5] Haggerty C Pitt E Siliciano R The latent reservoir for HIV in resting cells and other viral reservoirs during chronic infection: insight from treatment and treatment-interruption trials Curr Opin HIV AIDS 2006 1 62 68 10.1097/01.COH.0000191897.78309.70 19372786

[B6] Tabib A Leroux C Mornex JF Loire R Accelerated coronary atherosclerosis and arteriosclerosis in young human immunodeficiency-virus-positive patients Coron Artery Dis 2000 11 41 46 10.1097/00019501-200002000-00008 10715805

[B7] Currier JS Taylor A Boyd F Dezii CM Kawabata H Burtcel B Maa JF Hodder S Coronary heart disease in HIV-infected individuals J Acquir Immune Defic Syndr 2003 33 506 512 10.1097/00126334-200308010-00012 12869840

[B8] Bozzette SA Ake CF Tam HK Chang SW Louis TA Cardiovascular and cerebrovascular events in patients treated for human immunodeficiency virus infection N Engl J Med 2003 348 702 710 10.1056/NEJMoa022048 12594314

[B9] Mu H Chai H Lin PH Yao Q Chen C Current update on HIV-associated vascular diseases and endothelial dysfunction World J Surg 2007 31 632 643 10.1007/s00268-006-0730-0 17372667

[B10] Crowe SM Westhorpe CLV Mukhamedova N Jawororowski A Sviridov D Burkinsky M The macrophage: the intersection between HIV infection and atherosclerosis J Leukoc Biol 2010 87 589 598 10.1189/jlb.0809580 19952353 PMC3085483

[B11] Joshi VV Pawel B Connor E Sharer L Oleske JM Morrison S Marin-Garcia J Arteriopathy in children with acquired immune deficiency syndrome Pediatr Pathol 1987 7 261 275 10.3109/15513818709177129 3684808

[B12] Paton P Tabib A Loire R Tete R Coronary artery lesions and human immunodeficiency virus infection Res Virol 1993 144 225 231 8356344 10.1016/s0923-2516(06)80033-6

[B13] Lorenz MW Stephan C Harmjanz A Staszewski S Buehler A Bickel M von Kegler S Ruhkamp D Steinmetz H Sitzer M Both long-term HIV infection and highly active antiretroviral therapy are independent risk factors for early carotid atherosclerosis Atherosclerosis 2008 196 720 726 10.1016/j.atherosclerosis.2006.12.022 17275008

[B14] Grunfeld C Delaney JA Wanke C Currier JS Scherzer R Biggs ML ien PC Shlipak MG Sidney S Polak JF O'Leary D Bacchetti P Kronmal RA Preclinical atherosclerosis due to HIV infection: carotid intimamedial thickness measurements from the FRAM study AIDS 2009 23 1841 1849 10.1097/QAD.0b013e32832d3b85 19455012 PMC3156613

[B15] Hsue PY Hunt PW Schnell A Kalapus SC Hoh R Ganz P Martin JN Deeks SG Role of viral replication, antiretroviral therapy, and immunodeficiency in HIV-associated atherosclerosis AIDS 2009 23 1059 1067 10.1097/QAD.0b013e32832b514b 19390417 PMC2691772

[B16] Vittecoq D Escaut L Chironi G Teicher E Monsuez JJ Andrejak M Simon A Coronary heart disease in HIV-infected patients in the highly active antiretroviral treatment era AIDS 2003 17 S70 S76 12870533 10.1097/00002030-200304001-00010

[B17] Currier JS Lundgren JD Carr A Klein D Sabin CA Sax PE Schouten JT Smieja M Epidemiological evidence for cardiovascular disease in HIV-infected patients and relationship to highly active antiretroviral therapy Circulation 2008 118 e29 e35 10.1161/CIRCULATIONAHA.107.189624 18566319 PMC5153327

[B18] Moreno PR Sanz J Fuster V Promoting mechanisms of vascular health J Am Coll Cardiol 2009 53 2315 2323 10.1016/j.jacc.2009.02.057 19539140

[B19] Hansson GK Inflammation, Atherosclerosis and coronary artery disease N Eng J Med 2005 352 1685 1695 10.1056/NEJMra043430 15843671

[B20] Galkina E Ley K Immune and inflammatory mechanisms of atherosclerosis Annu Rev Immunol 2009 27 165 197 10.1146/annurev.immunol.021908.132620 19302038 PMC2734407

[B21] Birx DL Redfield RR Tencer K Fowler A Burke DS Tosato G Induction of interleukin-6 during human immunodeficiency virus infection Blood 1990 76 2303 2310 2257304

[B22] Buonaguro L Barillari G Chang HK Bohan CA Kao V Morgan R Gallo RC Ensoli B Effects of the human immunodeficiency virus type 1 Tat protein on the expression of inflammatory cytokines J Virol 1992 66 7159 7167 1279199 10.1128/jvi.66.12.7159-7167.1992 PMC240407

[B23] Scala G Ruocco MR Ambrosino C Mallardo M Giordano V Baldassare F Dragonetti E Quinto I Venuta S The expression of the interleukin 6 gene is induced by the human immunodeficiency virus 1 TAT protein J Exp Med 1994 179 961 971 10.1084/jem.179.3.961 8113688 PMC2191426

[B24] Barqasho B Nowak P Tjernlund A Kinloch S Goh LE Lampe F Fisher M Andersson J Sönnerborg A QUEST study group Kinetics of plasma cytokines and chemokines during primary HIV-1 infection and after analytical treatment interruption HIV Med 2009 10 94 102 10.1111/j.1468-1293.2008.00657.x 19200172

[B25] Birdsall HH Porter WJ Green DM Rubio J Trial J Rossen RD Impact of fibronectin fragments on the transendothelial migration of HIV-infected leukocytes and the development of subendothelial foci of infectious leukocytes J Immunol 2004 173 2746 2754 15294993 10.4049/jimmunol.173.4.2746

[B26] Tilton JC Johnson AJ Luskin MR Manion MM Yang J Adelsberger JW Lempicki RA Hallahan CW McLaughlin M Mican JM Metcalf JA Iyasere C Connors M Diminished production of monocyte proinflammatory cytokines during human immunodeficiency virus viremia is mediated by type I interferons J Virol 2006 80 11486 11497 10.1128/JVI.00324-06 17005663 PMC1642603

[B27] Zucker-Franklin D Grusky G Marcus A Transformation of monocytes into "fat" cells Lab Invest 1978 38 620 628 642458

[B28] Mujawar Z Rose H Morrow MP Pushkarsky T Dubrovsky L Mukhamedova N Fu Y Dart A Orenstein JM Bobryshev YV Bukrinsky M Svirdov D Human immunodeficiency virus impairs reverse cholesterol transport from macrophages PLoS Biol 2006 4 e365 10.1371/journal.pbio.0040365 17076584 PMC1629034

[B29] Bukrinsky M Sviridov D HIV and cardiovascular disease: contribution of HIV-infected macrophages to development of atherosclerosis PLoS Med 2007 4 e43 10.1371/journal.pmed.0040043 17411317 PMC1796648

[B30] Huang MB Khan M Garcia-Barrio M Powell M Bond VC Apoptotic effects in primary human umbilical vein endothelial cell cultures caused by exposure to virion-associated and cell membrane-associated HIV-1 gp120 J Acquir Immune Defic Syndr 2001 27 213 221 11464139 10.1097/00126334-200107010-00001

[B31] Ullrich CK Groopman JE Ganju RK HIV-1 gp120- and gp160-induced apoptosis in cultured endothelial cells is mediated by caspases Blood 2000 96 1438 1442 10942389

[B32] Park IW Ullrich CK Schoenberger E Ganju RK Groopman JE HIV-1 Tat induces microvascular endothelial apoptosis through caspase activation J Immunol 2001 167 2766 2771 11509621 10.4049/jimmunol.167.5.2766

[B33] Asahara T Murohara T Sullivan A Silver M Van der Zee R Li T Witzenbichler B Schatterman G Isler JM Isolation of putative progenitor endothelial cells for angiogenesis Science 1997 275 964 967 10.1126/science.275.5302.964 9020076

[B34] Asahara T Isner JM Endothelial progenitor cells for vascular regeneration J Hematother Stem Cell Res 2002 11 171 178 10.1089/152581602753658385 11983091

[B35] Asahara T Kawamoto A Endothelial progenitor cells for postnatal vasculogenesis Am J Physiol Cell Physiol 2004 287 C572 C579 10.1152/ajpcell.00330.2003 15308462

[B36] Ergun S Hohn HP Kilic N Singer BB Tilki D Endothelial and hematopoietic progenitor cells (EPCs and HPCs): hand in hand fate determining partners for cancer cells Stem Cell Rev 2008 4 169 177 10.1007/s12015-008-9028-y 18607782

[B37] Klein D Hohn HP Kleff V Tilkl D Ergun S Vascular wall-resident stem cells Histol Histopathol 2010 25 681 689 20238305 10.14670/HH-25.681

[B38] Pasquinelli G Pacilli A Alviano F Foroni L Ricci F Valente S Orrico C Lanzoni G Buzzi M Tazzari L Pagliaro PP Stella A Bagnara GP Multidistrict human mesenchymal vascular cells: pluripotency and stemness characteristics Cytotherapy 2010 12 275 287 10.3109/14653241003596679 20230218

[B39] Scadden DT Zeira M Woon A Wang Z Schieve L Ikeuchi K Lim B Groopman JE Human immunodeficiency virus infection of human bone marrow stromal fibroblasts Blood 1990 76 317 322 1695109

[B40] Wang L Mondal D La Russa VF Agrawal KC Suppression of clonogenic potential of human bone marrow mesenchymal stem cells by HIV type 1: putative role of HIV type 1 tat protein and inflammatory cytokines AIDS Res Hum Retroviruses 2002 18 917 931 10.1089/088922202760265597 12230935

[B41] Cotter EJ Ip HSM Powderly WG Doran PP Mechanism of HIV protein induced modulation of mesenchymal stem cell osteogenic differentiation BMC Musculoskeletal Disorders 2008 9 33 10.1186/1471-2474-9-33 18366626 PMC2330047

[B42] Cotter EJ Chew N Powderly WG Doran PP HIV type 1 alters mesenchymal stem cell differentiation potential and cell phenotype ex-vivo AIDS Res Hum Retrov 2011 27 187 199 10.1089/aid.2010.0114 20929345

[B43] Canque B Marandin A Rosenzwajg M Louache F Vainchenker W Gluckman JC Susceptibility of human bone marrow stromal cells to human immunodeficiency virus (HIV) Virology 1995 208 779 783 10.1006/viro.1995.1211 7747451

[B44] Pasquinelli G Tazzari PL Vaselli C Foroni L Buzzi M Storci G Alvaino F Ricci F Bonafè M Orrico C Bagnara GP Stella A Conte R Thoracic aortas from multiorgan donors are suitable for obtaining resident angiogenic mesenchymal stromal cells Stem Cells 2007 25 1627 1634 10.1634/stemcells.2006-0731 17446560

[B45] Alviano F Fossati V Marchionni C Arpinati M Bonsi L Franchina M Lanzoni G Cantoni S Cavallini C Bianchi F Tazzari PL Pasquinelli G Foroni L Ventura C Grossi A Bagnara GP Term amniotic membrane is a high throughput source for multipotent mesenchymal stem cells with the ability to differentiate into endothelial cells in vitro BMC Dev Biol 2007 7 11 10.1186/1471-213X-7-11 17313666 PMC1810523

[B46] Oswald J Boxberger S Jorgensen B Feldmann S Ehringer G Bornhauser M Werner C Mesenchymal stem cells can be differentiated into endothelial cells in vitro Stem Cells 2004 22 377 384 10.1634/stemcells.22-3-377 15153614

[B47] Chelucci C Federico M Guerriero R Mattia G Casella I Pelosi E Testa U Mariani G Hassan HJ Peschle C Productive human immunodeficiency virus-1 infection of purified megakaryocytic progenitors/precursors and maturing megakaryocytes Blood 1998 91 1225 1234 9454752

[B48] Guo L Heinzinger NK Stevenson M Schopfer LM Salhany JM Inhibition of gp120-CD4 interaction and human immunodeficiency virus type 1 infection in vitro by pyridoxal 5'-phosphate Antimicrob Agents Chemother 1994 38 2483 2487 7530934 10.1128/aac.38.10.2483 PMC284769

[B49] Gibellini D De Crignis E Ponti C Cimatti L Borderi M Tschon M Giardino R Re MC HIV-1 triggers apoptosis in primary osteoblasts and HOBIT cells through TNFalpha activation J Med Virol 2008 80 1507 1514 10.1002/jmv.21266 18649336

[B50] Gibellini D Vitone F Schiavone P Ponti C La Placa M Re MC Quantitative detection of human immunodeficiency virus type 1 (HIV-1) proviral DNA in peripheral blood monuclear cells by SYBR green real-time PCR technique J Clin Virol 2004 29 282 289 10.1016/S1386-6532(03)00169-0 15018857

[B51] Lehrman G Hogue IB Palmer S Jennings C Spina CA Wiegand A Landay AL Coombs RW Richman DD Mellors JW Coffin JM Bosch RJ Margolis DM Depletion of latent HIV-1 infection in vivo: a proof of concept study Lancet 2005 366 549 555 10.1016/S0140-6736(05)67098-5 16099290 PMC1894952

[B52] O'Doherty U Swiggard WJ Jeyakumar D McGain D Malim MH A sensitive, quantitative assay for human immunodeficiency virus type 1 integration J Virol 2002 76 10942 10500 10.1128/JVI.76.21.10942-10950.2002 12368337 PMC136638

[B53] Kim DW Real time quantitative PCR Exp Mol Med 2001 33 101 109 11708318

[B54] De Gemmis P Lapucci C Bertelli M Tognetto A Fanin E Vettor R Pagano C Pandolfo M Fabbri A A real-time PCR approach to evaluate adipogenic potential of amniotic fluid-derived human mesenchymal stem cells Stem Cells Dev 2006 15 719 726 10.1089/scd.2006.15.719 17105407

[B55] Montaln H Schichor C Lah TT Human mesenchymal stem cells and their use in cell-based terapies Cancer 2010 116 2519 2530 10.1002/cncr.25056 20301117

[B56] Chamberlain G Fox J Ashton B Middleton J Mesenchymal stem cells: their phenotype, differentiation capacity, immunological features, and potential for homing Stem Cells 2007 25 2739 2749 10.1634/stemcells.2007-0197 17656645

[B57] Tavian M Zheng B Oberlin E Crisan M Sun B Huard J Peault B The vascular wall as a source of stem cells Ann N Y Acad Sci 2005 1044 41 50 10.1196/annals.1349.006 15958696

[B58] Zengin E Chalajour F Gehling UM Ito WD Treede H Lauke H Weil J Reichenspumer H Kilic N Ergun S Vascular wall resident progenitor cells: a source for postnatal vasculogenesis Development 2006 133 1543 1551 10.1242/dev.02315 16524930

[B59] Passman JN Dong XR Wu SP Maguire CT Hogan KA Bautch VL Majesky MW A sonic hedgehog signaling domain in the arterial adventitia supports resident Sca1+ smooth muscle progenitor cells Proc Natl Acad Sci USA 2008 105 9349 9354 10.1073/pnas.0711382105 18591670 PMC2453724

[B60] Abedin M Tintut Y Demer LL Mesenchymal stem cells and the artery wall Circul Res 2004 95 671 676 10.1161/01.RES.0000143421.27684.12 15459088

[B61] Klein D Hurley LB Quesenberry CP Jr Sidney S Do protease inhibitors increase the risk for coronary heart disease in patients with HIV-1 infection? J Acquir Immune Defic Syndr 2002 30 471 477 10.1097/00126334-200208150-00002 12154337

[B62] Moses A Nelson J Bagby G The influence of human immunodeficiency virus-1 on hematopoiesis Blood 1998 91 1479 1495 9473211

[B63] Chirmule N Pahwa S Envelope glycoproteins of human immunodeficiency virus type 1:profound influences on immune functions Microbiol Rev 1996 60 386 406 8801439 10.1128/mr.60.2.386-406.1996 PMC239449

[B64] Re MC Zauli G Gibellini D Furlini G Ramazzotti E Monari P Ranieri S Capitani S La Placa M Uninfected haematopoietic progenitor (CD34+) cells purified from the bone marrow of AIDS patients are committed to apoptotic cell death in culture AIDS 1993 7 1049 1055 10.1097/00002030-199308000-00004 7691085

[B65] Banda NK Bernier J Kurahara DK Kurrle R Haigwood N Sekaly RP Finkel TH Crosslinking CD4 by human immunodeficiency virus gp120 primes T cells for activation-induced apoptosis J Exp Med 1992 176 1099 1106 10.1084/jem.176.4.1099 1402655 PMC2119378

[B66] Lawson VA Silburn KA Gorry PR Paukovic G Purcell DF Greenway AL McPhee DA Apoptosis induced in synchronized human immunodeficiency virus type 1-infected primary peripheral blood mononuclear cells is detected after the peak of CD4+ T-lymphocyte loss and is dependent on the tropism of the gp120 envelope glycoprotein Virology 2004 327 70 82 10.1016/j.virol.2004.06.012 15327899

[B67] Nacher M Serrano S Gonzalez A Hernandez A Marinoso ML Vilella R Hinarejos P Diez A Aubia J Osteoblasts in HIV-infected patients: HIV-1 infection and cell function AIDS 2001 15 2239 2243 10.1097/00002030-200111230-00004 11698696

[B68] Adachi A Koenig S Gendelman HE Daugherty D Gattoni-Celli S Fauci AS Martin MA Productive, persistent infection of human colorectal cell lines with human immunodeficiency virus J Virol 1987 61 209 213 3640832 10.1128/jvi.61.1.209-213.1987 PMC255241

[B69] Nottet H Janse I De Graaf L Bakker LJ Visser MR Verhoef J Infection of epithelial cell line HEp-2 with human immunodeficiency virus type 1 is CD4 dependent J Med Virol 1993 40 39 43 10.1002/jmv.1890400109 8515246

[B70] Gerber HP McMurtrey A Kowalski J Yan M Keyt BA Dixit V Ferrara N Vascular endothelial growth factor regulates endothelial cell survival through the phosphatidylinositol 3-kinase/Akt signal transduction pathway. Requirement for Flk-1/KDR activation J Biol Chem 1998 273 30336 30343 (1998) 10.1074/jbc.273.46.30336 9804796

[B71] Grünewald FS Prota AE Giese A Ballmer-Hofer K Structure-function analysis of VEGF receptor activation and the role of coreceptors in angiogenic signaling Biochim Biophys Acta 2010 1804 567 580 19761875 10.1016/j.bbapap.2009.09.002

[B72] Chawla A Lazar MA Peroxisome proliferator and retinoid signaling pathways co-regulate preadipocyte phenotype and survival Proc Natl Acad Sci USA 1994 91 1786 1790 10.1073/pnas.91.5.1786 8127882 PMC43248

[B73] Lehmann JM Moore LB Smith-Oliver TA Wilkison WO Willson TM Kliewer SA An antidiabetic thiazolidinedione is a high affinity ligand for peroxisome proliferator-activated receptor gamma (PPAR gamma) J Biol Chem 1995 270 12953 12956 10.1074/jbc.270.22.12953 7768881

[B74] MacDougal OA Lane MD Adipocyte differentiation. When precursors are also regulators Curr Biol 1995 5 618 621 10.1016/S0960-9822(95)00125-4 7552171

[B75] Mandrup S Lane MD Regulating adipogenesis J Biol Chem 1997 272 5367 5370 10.1074/jbc.272.9.5367 9102400

[B76] Wu Z Xie Y Bucher NL Farmer SR Conditional ectopic expression of C/EBP beta in NIH-3T3 cells induces PPAR gamma and stimulates adipogenesis Genes Dev 1995 9 2350 2363 10.1101/gad.9.19.2350 7557387

[B77] Barak Y Nelson MC Ong ES Jones YZ Ruiz-Lozano P Chien KR Koder A Evans RM PPAR gamma is required for placental, cardiac, and adipose tissue development Mol Cell 1999 4 585 595 10.1016/S1097-2765(00)80209-9 10549290

[B78] Cotter EJ Malizia AP Chew N Powderly WG Doran PP HIV proteins regulate bone marker secretion and transcription factor activity in cultured human osteoblasts with consequent potential implications for osteoblast function and development AIDS Res Hum Retroviruses 2007 23 1521 1530 10.1089/aid.2007.0112 18160010

[B79] Cotter EJ Mallon PW Doran PP Is PPARγ a prospective player in HIV-1 associated bone disease? PPAR Res 2009 2009 421376 19325916 10.1155/2009/421376 PMC2659551

[B80] Popik W Hesselgesser JE Pitha PM Binding of human immunodeficiency virus type 1 to CD4 and CXCR4 receptors differentially regulates expression of inflammatory genes and activates the MEK/ERK signaling pathway J Virol 1998 72 6406 6413 9658081 10.1128/jvi.72.8.6406-6413.1998 PMC109793

[B81] Folkman J Watson K Ingber D Hanahan D Induction of angiogenesis during the transition from hyperplasia to neoplasia Nature 1989 339 58 61 10.1038/339058a0 2469964

[B82] Risau W Mechanisms of angiogenesis Nature 1997 386 671 674 9109485 10.1038/386671a0

[B83] Nikolova G Strilic B Lammert E The vascular niche and its basement membrane Trends Cell Biol 2007 17 19 25 10.1016/j.tcb.2006.11.005 17129728

[B84] Lafeuillade A Alessi MC Poizot-Martin I Boyer-Neumann C Zandotti C Quilchini R Aubert L Tamalet C Juhan-Vague I Gastaut JA Endothelial cell dysfunction in HIV infection J Acquir Immune Defic Syndr 1992 5 127 131 1531074

[B85] Toulon P Lamine M Ledjev I Guez T Holleman ME Sereni D Sicard D Heparin cofactor II deficiency in patients infected with the human immunodeficiency virus Thromb Haemost 1993 70 730 735 8128426

[B86] Dallasta LM Pisarov LA Esplen JE Werley JV Moses AV Nelson JA Achim CL Blood-brain barrier tight junction disruption in human immunodeficiency virus-1 encephalitis Am J Pathol 1999 155 1915 1927 10.1016/S0002-9440(10)65511-3 10595922 PMC1866950

[B87] Gibellini D Zauli G Re MC Milani D Furlini G Caramelli E Capitani S La Placa M Recombinant human immunodeficiency virus type-1 (HIV-1) Tat protein sequentially up-regulates IL-6 and TGF-beta 1 mRNA expression and protein synthesis in peripheral blood monocytes Br J Haematol 1994 88 261 267 10.1111/j.1365-2141.1994.tb05016.x 7803268

[B88] Hoffman FM Chen P Incardona F Zidovetzki R Hinton DR HIV-1 tat protein induces the production of interleukin-8 by human brain-derived endothelial cells J Neuroimmunol 1999 94 28 39 10.1016/S0165-5728(98)00198-2 10376933

[B89] Kanmogne GD Primeaux C Grammas P Induction of apoptosis and endothelin-1 secretion in primary human lung endothelial cells by HIV-1 gp120 proteins Biochem Biophys Res Commun 2005 333 1107 1115 10.1016/j.bbrc.2005.05.198 15979050

[B90] Clouse KA Cosentino LM Weih KA Pyle SW Robbins PB Hochstein HD Natarajan V Farrar WL The HIV-1 gp120 envelope protein has the intrinsic capacity to stimulate monokine secretion J Immunol 1991 147 2892 2901 1918997

[B91] El-Sadr WM Mullin C Carr A Gibert C Rappoport C Visnegarwala F Grunfeld C Raghavan SS Effects of HIV disease on lipid, glucose and insulin levels: results from a large antiretroviral-naive cohort HIV Med 2005 6 114 121 10.1111/j.1468-1293.2005.00273.x 15807717

[B92] Rose H Hoy J Woolley I Tchoua U Bukrinsky M Dart A Sviridov D HIV infection and high density lipoprotein metabolism Atherosclerosis 2008 199 79 86 10.1016/j.atherosclerosis.2007.10.018 18054941 PMC2518204

[B93] Qiao JH Mertens RB Fishbein MC Geller SA Cartilaginous metaplasia in calcified diabetic peripheral vascular disease: morphologic evidence of enchondral ossification Hum Pathol 2003 34 402 407 10.1053/hupa.2003.72 12733123

[B94] Tang T Boyle JR Dixon AK Varty K Inflammatory abdominal aortic aneurysms Eur J Vasc Endovasc Surg 2005 29 353 362 15749035 10.1016/j.ejvs.2004.12.009

